# Can Residual Inhibition Predict the Success of Sound Enrichment Treatment for Tinnitus?

**DOI:** 10.1002/brb3.70083

**Published:** 2024-10-08

**Authors:** Eser Sendesen, Didem Turkyilmaz

**Affiliations:** ^1^ Department of Audiology Hacettepe University Ankara Turkey

**Keywords:** residual inhibition, sound enrichment, tinnitus, tinnitus pitch

## Abstract

**Objectives:**

The objective of this study was to investigate whether residual inhibition (RI), which provides information on the relationship between tinnitus and increased spontaneous activity in the auditory system, is a predictor for the success of sound enrichment treatment.

**Design:**

Tinnitus patients were divided into two groups based on whether RI was achieved (RI+) or not (RI−). All participants underwent sound enrichment. Psychosomatic measures (for tinnitus severity, discomfort, attention deficit and sleep difficulties), Tinnitus Handicap Inventory (THI), minimum masking level (MML), and tinnitus loudness level (TLL) results were compared before and at 1, 3, and 6 months after treatment.

**Study sample:**

Sixty‐seven chronic tinnitus patients were divided into two groups based on whether RI was achieved (RI+) or not (RI−). There were 38 patients in the RI+ group and 29 in the RI− group.

**Results:**

There was a statistically significant difference between the groups in psychosomatic measures, THI, MML and TLL scores at the post‐treatment 6 months after treatment (*p* <.05). There was a statistically significant decrease in psychosomatic measures, THI, MML and TLL scores during the treatment period in the RI+ group, but not in the RI− group.

**Conclusion:**

RI may predict the prognosis of tinnitus treatments used in clinics to reduce the spontaneous firing rate of neurons in the central auditory system, and that RI positivity may be a predictor of treatment success in sound enrichment.

## Graphical abstract

Effect of residual inhibition status on minimum masking level scores

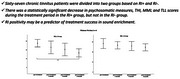

## 1 Introduction

Approximately 15% of adults suffer from chronic tinnitus (Biswas et al. 2022). Tinnitus can result in significant problems such as insomnia (Schecklmann et al. 2015), depression (Salazar et al. 2019), and cognitive issues (Clarke et al. 2020,Gürses et al. 2018). Studies on treatment methods to suppress tinnitus and solve these problems are still ongoing.

One tinnitus treatment is sound enrichment. This method uses the sound complex to suppress tinnitus in the hearing loss frequency range (Vanneste et al. 2013). Studies have shown that keeping animals in an acoustic environment rich in high frequencies after noise trauma can prevent maladaptive central auditory system reorganization (Noreña and Eggermont 2005). Another study found that when high‐frequency sound enrichment was used on animals exposed to noise trauma, there was no difference in the level of spontaneous activity in the central auditory system compared to animals with normal hearing (Noreña and Eggermont 2006).

In a previous study, tinnitus patients' clinical and electrophysiological responses to bandpass‐filtered music in the hearing loss region were examined (Vanneste et al. 2013). No reduction in tinnitus was observed. However, Vanneste et al. (2013) did not perform an assessment of the tinnitus etiology in their study, although they shared the participants' hearing thresholds. Sound enrichment may even increase the perception of tinnitus in some patients with hearing loss whose etiology is not hearing loss (Kaltenbach 2006). As mentioned, sound enrichment primarily improves the central auditory system affected by hearing loss, as peripheral auditory input can reduce hyperactivity caused by hearing loss. But in other etiologies, it can stimulate brainstem nuclei associated with the tinnitus etiology via ephaptic interactions, increasing their activity and tinnitus (Levine and Oron 2015)

Increased spontaneous activity in the auditory system may cause tinnitus (Galazyuk et al. 2019; Roberts 2007). Previous studies have linked residual inhibition (RI) to increased auditory spontaneous activity (Galazyuk et al. 2019; Roberts 2007; Schoisswohl et al. 2021). RI describes the change in tinnitus loudness after narrow band noise with a pitch‐matched center frequency: positive if narrow band noise decreases tinnitus perception and negative if it increases or does not change (Roberts 2007). Changes in tinnitus loudness may be linked to auditory system spontaneous activity (Hu et al. 2021). Positive RI reduces tinnitus by suppressing peripheral auditory system spontaneous activity while negative RI suggests tinnitus is unrelated to auditory system spontaneous activity (Galazyuk et al. 2019; Roberts 2007). As a result, auditory input presented during RI will inherently suppress hyperactivity in the peripheral auditory system. If tinnitus perception decreases after suppression, this hyperactivity could be related to tinnitus. Hearing loss has been shown to be the leading cause of hyperactivity in the peripheral and central auditory system (Zhao et al. 2016). Thus, RI is an important variable in the study of hearing loss‐related tinnitus because it indicates whether the increase in spontaneous peripheral auditory system activity is linked to it (Hu et al. 2021).

According to animal studies, sound enrichment may only benefit tinnitus associated with hearing loss because it reduces peripheral auditory system hyperactivity (Noreña and Eggermont 2005, 2006; Sendesen and Turkyilmaz 2024). Conversely, RI can reveal whether hearing loss increases spontaneous peripheral auditory system activity and tinnitus (Galazyuk et al. 2019; Hu et al. 2021; Roberts 2007). Tinnitus was not caused by increased spontaneous activity in the peripheral auditory system due to hearing loss, so sound enhancement may not have suppressed it in Vanneste et al. (2013). When RI is considered one of the variations, although not the only one, success in sound enrichment treatment was achieved in our previous study (Sendesen and Turkyilmaz 2024). In addition, the preliminary study showthat in the short term (three months) RI status may have an effect on sound enrichment treatment (Sendesen 2023). The present study aimed to investigate whether RI alone, which provides information on the relationship between spontaneous activity increase and hearing loss, predicts the success of sound enrichment in reducing tinnitus. As a result, we divided the participants into two groups according to their RI status and applied sound enrichment to both. At the end of 6 months, we assessed time‐dependent changes in tinnitus psychoacoustic characteristics and questionnaire results within and between groups.

## 2 Material and Methods

### 2.1 Process and Participants

Ethical approval for this study was obtained from the Clinical Research Ethics Committee (GO23/285) .This study included patients with chronic tinnitus (more than 6 months) who previously applied to our clinic, met the inclusion criteria, and consented to participate. Based on their medical history, radiological images, and audiological evaluation results, patients with potential organic problems (such as otosclerosis, demyelinating diseases, and otitis media) related to the etiology of tinnitus or diagnosed psychiatric problems or patients who had abnormal external or middle ear status upon otoscopic and tympanometric examination or with any significant air‐bone gaps (i.e., exceeding 10 dB) were additionally excluded from the study. A calibrated Interacoustics AC‐40 audiometer, TDH‐39P headphones for testing 0.125–8 kHz, Sennheiser HDA200 headphones for testing 9–20 kHz, and a Radioear B‐71 bone vibrator were used for pure tone audiometry measures. Tinnitus patients with hearing thresholds greater than 90 dB in the 0.125–20 kHz range were excluded from the study to ensure that the individualized sound used for sound enrichment provided adequate input at all frequencies. Additionally, it was intended that the groups' hearing thresholds would be similar to present comparable levels of sound enrichment to both groups.

In conclusion, the study included 67 participants aged 21‐45 years who were previously applied to our clinic, and the data of these participants were analyzed retrospectively. Participants were then assigned to the RI+ or RI− group according to their RI status. The RI+ group had 38 participants (17 males and 21 females) ranging in age from 23 to 45. The RI− group (13 males and 16 females) included 29 individuals between the ages of 21 and 42. All participants had tonal tinnitus except for one participant in the RI+ group who had complex sounds (described as wind). Tinnitus psychoacoustic assessment, Tinnitus Handicap Inventory (THI), and psychosomatic measurement data obtained for all 1‐, 3‐, and 6‐month follow‐up examinations were analyzed.

### 2.2 Tinnitus Psychoacoustic Assessment

TDH‐39P headphones were used below 8 kHz and Sennheiser HDA200 headphones above 8 kHz during tinnitus psychoacoustic evaluations. Ipsilateral ear refers to the affected ear in unilateral tinnitus, the ear with a louder perception in bilateral asymmetric tinnitus, and one of the two ears in bilateral symmetrical or head tinnitus. The contralateral ear is untested. To avoid patient confusion with tinnitus auditory stimuli, stimuli were presented at 30 dB SL (according to the hearing threshold of the relevant frequency in the participants' audiogram) from the contralateral ear for pitch matching. A two‐alternative forced selection procedure was used from 0.125 to 20 kHz. In each section, participants were shown two frequency pairs (one second each) and asked to choose the frequency that best matched their tinnitus pitch, whether tonal or complex. The first frequency pair was 0.5/4 kHz (Yakunina and Nam 2021). The patient's frequency and an octave higher or lower are next. If the patient chose 4 kHz from the first pair, they were presented 4/8 kHz. If the patient chose 0.5 kHz from the first pair, a 0.25/0.5 kHz pair presented. It was repeated in half‐octave increments until the final frequency was found. Participants chose the same tone frequency three times in each frequency pair to determine tinnitus pitch. Tinnitus pitch was obtained for all participants.

The psychoacoustic perception of tinnitus loudness is called the Tinnitus Loudness Level (TLL). The TLL was determined using the tinnitus pitch match frequency. Since the audiometer we used in this study allows intensity variations of 5 dB, that tone was presented in 5 dB steps using a two‐alternative forced selection procedure (Haider et al. 2020). The participant was presented with two tones (one second each) at that frequency, with one tone at the hearing threshold and the other at 5 dB SL. If the participant chose the higher level, then the 5 dB SL tone and a 10 dB SL tone were presented, and he/she was asked to choose which was closer in loudness to the tinnitus. The procedure was repeated in 5 dB SL steps until the TLL was determined. When the participant picked the same tone and intensity (even at the threshold level) three times, the TLL was determined. All participants' TLLs were obtained, and those whose tinnitus symptoms disappeared were set to 0 for statistical analysis.

The minimum masking level (MML) is the lowest level at which tinnitus first becomes inaudible during a task in which a narrowband noise is raised in intensity from 0 dB in 5 dB steps (each lasting 5 s). In bilateral symmetric tinnitus, participants were exposed to narrowband noise bilaterally. In the case of unilateral tinnitus, it was presented to the tinnitus ear. Participants were asked to raise their hands when their tinnitus became inaudible due to the auditory stimulus. MMLs were obtained from all participants, and the MML value of those whose tinnitus symptom disappeared was set to 0 for statistical analysis.

RI is a phenomenon that investigates the existence of tinnitus suppression following auditory stimulation. RI was determined by presenting narrowband noise with a center frequency matching the tinnitus perception at 10 dB SL above the MML. This stimulus was presented in the tinnitus ear if tinnitus was unilateral and binaurally if tinnitus was bilateral. Since previous studies have shown that the masking effect of the transmitted stimulus occurs sufficiently within 60 s, narrow band noise was presented for 60 s (Roberts 2007). Then, the participant was asked whether the level of tinnitus perception had decreased, remained the same, or increased. Finally, these findings were considered positive if the level of tinnitus perception decreased after the narrow band noise was turned off, and negative if it did not change or increased.

### 2.3 Tinnitus Questionnaire

The THI was used to assess the impact of tinnitus on the participants' daily lives (Aksoy, Firat, and Alpar 2007; Newman, Jacobson, and Spitzer 1996). It consists of 25 questions. Tinnitus' subjective psychological effects are evaluated based on patient reports. The THI assesses the functional, emotional, and catastrophic impacts of tinnitus. Responses are categorized as “Yes,” “Sometimes,” and “No,” with corresponding scores of 4, 2, and 0 points, respectively.

### 2.4 Psychosomatic Measurements

Visual Analog Scale (VAS) was used to perform psychosomatic measurements (subjectively perceived tinnitus severity, discomfort, attention deficit, and sleep difficulty). Tinnitus severity was defined as the level of tinnitus perception in the last week, tinnitus discomfort as the level of disturbance caused by tinnitus in the last week, attention deficit as distraction caused by tinnitus in the last week, and sleep difficulty as difficulty falling asleep due to tinnitus in the last week. Participants were told that on the VAS scale presented, 0 points mean “none,” 10 points mean “unbearable,” and intermediate emotions should be scored between 0 and 10. The score labels were presented in the native language.

### 2.5 Sound Enrichment Treatment

**FIGURE 1:**
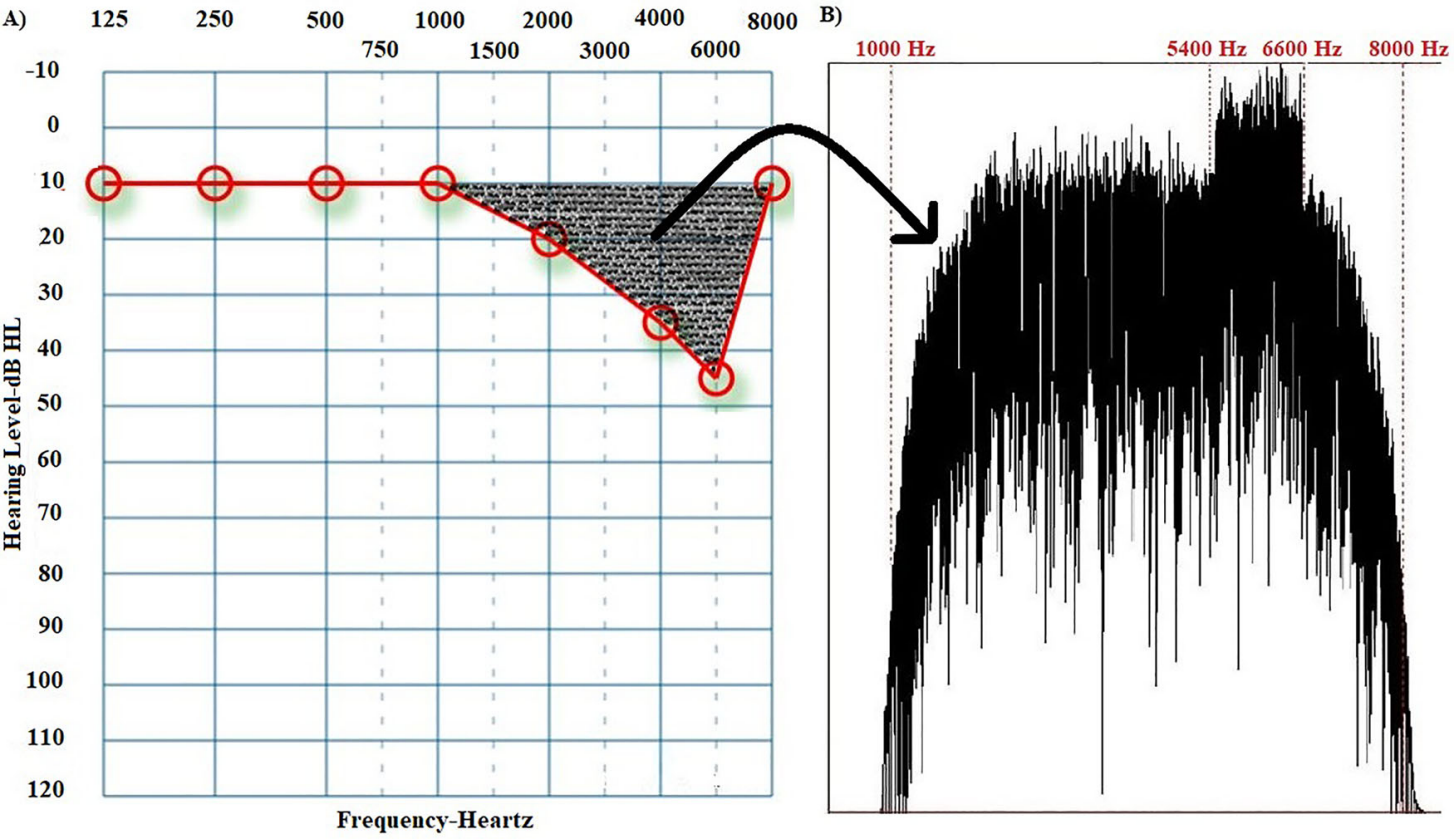
An example audiogram and spectrum of sound complex v3 created for treatment in accordance with the audiogram. (A) Audiogram of a patient with tinnitus at 6 kHz. The shaded area shows the spectrum range of the sound used for treatment. (B) The spectrum of the sound created for treatment (extra amplification in the range of 5.4–6.6 kHz, in addition to the frequency content in the range of 1–8 kHz)

Individual sound enrichment stimuli were created to suppress tinnitus with frequencies in the range of hearing loss (Vanneste et al. 2013). The Praat program created a sound complex from patient audiograms. Amplitude envelope fits hearing loss configuration. Its spectrum includes hearing loss frequencies and peaks at tinnitus pitch. First, a Gaussian distribution generated white noise with a sampling frequency of 44,100 Hz. This white noise was filtered with a band‐pass filter in the patient's hearing loss frequency range (Figure 1 shows that the range of hearing loss is 1–8 kHz, so the band‐pass filter range is 1–8 kHz). After determining the frequency ranges in band‐pass filtering, a smoothing parameter (a parameter that determines the degree of sharpness of the applied filter) was used to ensure that the targeted sound was appropriate for the hearing loss configuration while generating the filtering parameters. The values of this parameter vary from 1 to 100. A value of 1–10 is considered low, while a score of 25–100 is considered high (Albert, Cangemi, and Grice 2018). To calculate this value, we focused on the frequency ranges where hearing loss increased significantly (≥ 20 dB), thus revealing the hearing loss configuration. If the difference between adjacent hearing thresholds was large, a low smoothing was applied; if the difference was slight, a proportionally high smoothing was used (for example, in Figure 1, there is a 10‐dB difference in hearing thresholds in the 1‐ to 2kHz frequency range. Since this difference is not significant [≥ 20 dB] or even very close to 0, the smoothing parameter value was set to 80). Lastly, a sound complex v1 with spectral content was created to compensate for hearing loss. Another white noise was generated and band‐pass‐filtered at 10% below and above the patient's tinnitus pitch. Thus, sound complex v2 was created. The sound complex v2 was designed to reduce spontaneous activity, which was highest at the tinnitus pitch according to the discordant theory (Jastreboff 2004), by using a sound complex with the highest amplitude at the tinnitus pitch. Since sound enrichment suppresses spontaneous activity, we think it is important to increase the sound complex's amplitude in the tinnitus pitch area, where spontaneous activity is the highest. Finally, an individualized sound (sound complex v3) was created by combining sound complex v2 with sound complex v1 in mono. Figure 1 shows an example audiogram and the spectrum of the sound complex v3 based on this audiogram.

In the first month of treatment, participants were asked to listen to their individualized sounds with headphones for 6 h a day (without the need to focus on the sound), continuously or in parts, at a maximum intensity higher than the tinnitus intensity without discomfort (the highest volume level on the smartphone that suppresses tinnitus but does not disturb the participant). They used smartphones to listen to sounds for cost‐effectiveness and accessibility. From 1 to 3 months, listening time was reduced to 3 h and sound intensity was reduced to just above the tinnitus suppression level (one volume level above the minimum volume level that suppresses tinnitus on a smartphone). From 3–6 months, listening time was reduced to 2 h, and sound intensity was reduced to just below tinnitus (one volume level below the minimum required to mask tinnitus on a smartphone). The intensity level of the individualized sounds was explained and shown (the individualized sound uploaded to the participant's smartphone was played through headphones. Then, based on the participant's feedback, the required intensity level of the perceptually individualized sound was adjusted according to the process) to the participant prior to treatment and at the first‐ and third‐month follow‐up appointments.

### 2.6 Statistical Methods

The G*Power program was used to calculate the sample size for the study. Based on the mean and standard deviation values from the groups in the pilot study (Sendesen 2023) this study should have 21 participant in each group, with a 5% type I error level and a 95% minimum power to detect a significant difference. The data were evaluated using the SPSS version 25.0 package program (IBM Inc., Armonk, NY, USA). Time‐dependent changes (four phases of pre and post‐treatment 1,3 and 6 months) of psychosomatic measurements, THI, MML, and TLL scores within and between groups (RI+ and RI‐ groups were considered as between‐subjects factors) were analyzed using Repeated Measures ANOVA. Tukey post hoc tests used for multiple comparisons. Independent samples t‐test was used for age, hearing thresholds pre‐treatment psychosomatic measurements, THI, MML, and TLL scores. For all methods, a *p* value of .05 was considered statistically significant.

## 3 Results

### 3.1 Descriptive Statistics

Age distribution of the groups reveals that the mean age of the RI+ group was 31.18 ± 6.75 years. The mean age of the RI− group was 33.41 ± 7.37 years. Age did not differ significantly between groups (*t*(65) = .56, *p* = .58). At the end of the sixth month following sound enrichment treatment, 11 participants in the RI+ group no longer experienced tinnitus. In contrast, all participants in the RI− group continued to experience tinnitus. Table 1 shows the general tinnitus characteristics, and Table 2 shows the hearing thresholds and statistical evaluation results of the RI+ and RI− groups.

**TABLE 1:** General tinnitus characteristics.

	RI+ group	RI− group
Tinnitus location		
Right ear	11	7
Left ear	9	9
In the head/bilateral	18	13
Tinnitus frequency (kHz)	7.2 ± 0.7 (1–8)^a^	7.32 ± 0.6 (1–8)^a^
Tinnitus duration (months)	21.15 ± 8.3 (6−43)^a^	19.73 ± 11.36 (6–37)^a^
Hearing threshold at the tinnitus frequency	49.34 ± 7.27	47.89 ± 8.13

Abbreviation: RI, residual inhibition.

^a^ Minimum‐maximum value.

**TABLE 2:** Hearing thresholds for the RI+ and RI− groups.

	RI+ group	RI− group	
Frequency (kHz)	Mean ± SD	Mean ± SD	*p* value^a^
0.125	13.05 ± 8.76	15.8 ± 10.57	.37
0.25	16.94 ± 12.14	15.92 ± 10.73	.77
0.5	20 ± 12.48	19.2 ± 12.88	.84
1	29.72 ± 13.87	27.12 ± 11.72	.51
2	35.27 ± 8.48	36.4 ± 10.55	.71
4	44.72 ± 11.56	42 ± 13.14	.48
8	46.94 ± 19.71	41.25 ± 17.12	.32
10	50 ± 30.72	54.37 ± 24.99	.61
12	60.45 ± 17.38	63 ± 19.74	.75
16	64.54 ± 15.4	68.50 ± 12.48	.79
20	70 ± 10.95	71.5 ± 9.44	.74

Abbreviations: RI, residual inhibition; SD, standard deviation.

^a^ Independent sample *t*‐test.

### 3.2 Tinnitus Loudness and Minimum Masking Level

Table 3 shows the TLL and MML scores of the participants during treatment, as well as the statistical comparison between the groups. While TLL scores did not differ statistically (*t*(65) = .53, *p* = .61) between groups in pre‐treatment, there was a statistically significant difference between groups for time‐dependent change (*F*(1,65) = 15.45, *p* < .001, *r* = .51). TLL decreased statistically significantly in the RI+ group during the treatment process (*F*(3,148) = 14.85, *p* < .001, *r* = .65). The Tukey post hoc test showed a statistically significant difference between pre‐ and post‐treatment sixth month (*p* < .001). No statistically significant difference was observed in the RI− group (*F*(3,112) = 1.62, *p* = .74, *r* = .18) during the treatment process.

**TABLE 3:** Pre‐ and post‐treatment TLL, MML, and THI scores for the RI+ and RI− groups.

	RI+ group	RI− group	RI+ group	RI− group	RI+ group	RI− group
	Tinnitus loudness level		Minimum masking level		Tinnitus handicap inventory	
Time	Mean ± SD	Mean ± SD	Mean ± SD	Mean ± SD	Mean ± SD	Mean ± SD
Pre‐treatment	*64.5 ± 4.9	65.7 ± 7.3	57.3 ± 3.1	57.5 ± 5.8	*52.32 ± 21.39	52.58 ± 16.93
1 Months	*55.1 ± 8.9	61.2 ± 5.1	*42.4 ± 6.8	54.3 ± 4.1	34.08 ± *16.49	50.32 ± 15.51
3 Months	49.7 ± 11.3	60.1 ± 5.8	*40.8 ± 6.9	50.9 ± 5.2	24.87 ± 12.18	48.24 ± 14.01
6 Months	37.8 ± 5.8	59.2 ± 6.2	36.1 ± 3.7	49.8 ± 4.2	18.32 ± 3.01	47.47 ± 12.21
*p* value**	**< .001**	.78	**< .001**	.82	**< .001**	.66

*Note*: Bold *p* values show a statistically significant difference.

Abbreviations: RI, residual inhibition; SD, standard deviation.

*Tukey HSD, *p* < .001.

**Repeated measure ANOVA.

While MML scores did not differ statistically between groups in pre‐treatment (*t*(65) = .83, *p* = .41), there was a statistically significant difference between the groups for time‐dependent change MML scores (*F*(1,65) = 21.48, *p* < .001, *r* = .59). MML scores decreased statistically significantly in the RI+ group during the treatment process (*F*(3,148) = 46.03, *p* < .001, *r* = .85). The Tukey post hoc test showed a statistically significant difference between pre and post‐treatment sixth month (*p* < .001). No statistically significant difference was observed in the RI− group (*F*(3, 112) = 0.86, *p* = .76, *r* = .11) during the treatment process.

### 3.3 Tinnitus Handicap Inventory

Table 3 shows the THI scores of the participants during treatment, as well as the statistical comparison between the groups. While THI scores did not differ statistically between groups (*t*(65) = .42, *p* = .67) in pre‐treatment, there was a statistically significant difference between groups for time‐dependent change (*F*(1,65) = 15.04, *p* < .001, *r* = .50). THI scores in the RI+ group decreased statistically significantly during the treatment process (*F*(3,148) = 21.25, *p* <.001, *r* = .72). The Tukey post hoc test showed a statistically significant difference between pre‐ and post‐treatment sixth month (*p* <.001). No statistically significant difference was observed in the RI− group (*F*(3,112) = 1.24, *p* = .66, *r* = .14) during the treatment process.

### 3.4 Psychosomatic Measurements

Table 4 shows the VAS scores of the participants during treatment and the statistical comparison between the groups. While none of the psychosomatic measurements differed statistically (tinnitus severity t(65) = .56, *p* = .58, discomfort t(65) = .59, (*p* = .74), attention deficit (*t*(65) = .26, *p* = .79), sleep difficulty (*t*(65) = .54, *p* = .59) between groups in pre‐treatment, there was a statistically significant difference between groups for time‐dependent change (tinnitus severity F(1,65) = 20.95, *p* < .001, *r* = .58), discomfort (*F*(1,65) = 31.23, *p* < .001, *r* = .67), attention deficit (*F*(1,65) = 28.32, *p* < .001, *r* = .55), sleep difficulty (*F*(1,65) = 18.71, *p* < .001, *r* = .55). VAS scores in the RI+ group decreased statistically significantly during the treatment process (tinnitus severity, F(3,148) = 41.23, *p* <.001, *r* = .67), discomfort (*F*(3,148) = 38.73, *p* <.001, *r* = .71), attention deficit (*F*(3,148) = 42.18, *p* <.001, *r* = .68), and sleep difficulty (*F*(3,148) = 39.42, *p* <.001, *r* = .73). The Tukey post hoc test showed a statistically significant difference between pre‐ and post‐treatment six month for tinnitus severity (*p* < .001), discomfort (*p* <.001), attention deficit (*p* <.001), and sleep difficulty (*p* <.001) according to post hoc analysis. No statistically significant difference was observed in the RI− group (tinnitus severity, F(3,112) = 3.22, *p* = .27, *r* = .12; discomfort, F(3,112) = 2.75, *p* = .35, *r* = .13; attention deficit, F(3,112) = 1.29, *p* = .44, *r* = .14; sleep difficulty, F(3,112) = 1.54, *p* = .41, *r* = .11) during the treatment process.

**TABLE 4:** Pre‐ and post‐treatment VAS scores for the RI+ and RI− groups.

	RI+ group	RI− group	RI+ group	RI− group	RI+ group	RI− group	RI+ group	RI− group
	Tinnitus severity		Tinnitus discomfort		Attention deficit		Sleep difficulty	
Time	Mean ± SD	Mean ± SD	Mean ± SD	Mean ± SD	Mean ± SD	Mean ± SD	Mean ± SD	Mean ± SD
Pre‐treatment	*7.3 ± 1.9	7.8 ± 1.2	*7.3 ± 1.6	7.8 ± 1.5	*4.5 ± 1.8	4.9 ± 1.7	*6.2 ± 2.4	6.5 ± 2.5
1 Month	*6.4 ± 2.2	7.1 ± 1.9	*6.5 ± 2.1	7.4 ± 1.7	*3.8 ± 2.9	4.7 ± 1.6	*5.9 ± 1.6	6.1 ± 1.9
3 Months	*6.1 ± 1.6	6.7 ± 1.4	*5.9 ± 1.7	7.1 ± 1.2	*3.5 ± 1.1	4.2 ± 1.5	*5.3 ± 1.8	5.7 ± 2.4
6 Months	*4.3 ± 0.7	6.4 ± 1.1	*5.1 ± 0.7	6.8 ± 0.9	*2.7 ± 0.3	4.0 ± 1.3	*4.2 ± 0.8	5.6 ± 1.9
*p* value**	**<.001**	.17	**<.001**	.35	**<.001**	.44	**<.001**	.41

*Note*: Bold *p* values show a statistically significant difference.

Abbreviations: RI, residual inhibition; SD, standard deviation.

*Tukey HSD, *p* <.001.

**Repeated measure ANOVA.

## 4 Discussion

The main aim of this study was to investigate whether RI is a phenomenon that affects the success of sound enrichment. Based on the study's results, the THI, VAS, TLL, and MML scores showed a statistically significant reduction in the RI+ group when compared to the RI− group after sound enrichment. Furthermore, when within‐group analyses were performed, THI, VAS, TLL, and MML scores in the RI+ group decreased significantly by the end of the sixth month. In contrast, no change was observed in the RI− group. In addition, these findings were obtained at a similar sound enrichment level by keeping the hearing thresholds of both groups similar. Otherwise, variation in the peripheral auditory input level may cause different effects between groups in the central auditory system (Sheppard et al. 2020). Additionally, the age variable was controlled between groups due to its potential effect on the central auditory system (Ouda, Profant, and Syka 2015).

Hearing loss has previously been shown to increase the spontaneous activity of neurons in the peripheral and central auditory systems in animal and electrophysiological studies in humans (Herrmann and Butler 2021; Wang et al. 2020). It is additionally hypothesized that increased spontaneous activity is the basis of the pathophysiological mechanism of tinnitus associated with hearing loss (Jastreboff 2004; Seki and Eggermont 2003). In animal studies, spontaneous activity in the auditory system increased after artificially induced hearing loss, and maladaptive reorganization was observed in the central auditory system by altering its tonotopic organization (Noreña and Eggermont 2005). In a subsequent study conducted by the same researchers, sound enrichment was applied in the induced hearing loss frequency range. As a result, this study observed the functional organization in the central auditory system as the level of spontaneous activity and tonotopic organization in the auditory system returned to the level before hearing loss (Noreña and Eggermont 2006). Therefore, Vanneste et al. (2013) used sound enrichment as a potential treatment method for tinnitus patients, but tinnitus suppression was not achieved. The possibility that subjects in this study had etiologies of tinnitus not associated with hearing loss was not considered. Sound enrichment was found to be beneficial only in reducing increased spontaneous activity due to hearing loss in the animal studies mentioned previously. Sound therapy or sound enrichment may even be contraindicated in some tinnitus etiologies. Therefore, sound enrichment may be useful in tinnitus associated with increased spontaneous activity in the auditory system associated with hearing loss.

RI is a phenomenon that can temporarily reduce tinnitus perception for a short time after the presentation of an acoustic stimulus (Galazyuk et al. 2019; Schoisswohl et al. 2021). It plays an important role in reducing the spontaneous firing rates of neurons in the central auditory pathway, which encompasses the subcortical structures of the auditory system (Galazyuk, Voytenko, and Longenecker 2017; Schoisswohl et al. 2021). The electrophysiological brain activity of tinnitus patients after acoustic stimulation was investigated (King et al. 2021; Schoisswohl et al. 2021). After acoustic stimulation, RI‐positive tinnitus patients had lower activity in the central auditory system, whereas RI‐negative patients had no statistically significant change in spontaneous activity in the central auditory system. This result may suggest that RI may predict whether hyperactivity in the central auditory system can be reduced by acoustic stimulation.

As mentioned previously, studies have shown that sound enrichment reduces hyperactivity in the central auditory system following hearing loss and that even maladaptive changes in frequency organization caused by damage to the tuning curve of afferent nerve fibers are corrected (Noreña and Eggermont 2005, 2006). Sound enrichment may benefit tinnitus patients with increased spontaneous firing rates related to hearing loss. It has been observed that RI is often positive in tinnitus patients with hearing loss (Hu et al. 2021). Tinnitus caused by hearing loss, which induces neuroplastic changes in the auditory system, is more likely to involve changes in neural activity in the central auditory system (Herrmann and Butler 2021; Noreña and Eggermont 2005; Wang et al. 2020). In contrast, tinnitus caused by other etiologies affects a more complex neural network with neurons in both auditory and non‐auditory systems (Han et al. 2009; Shore, Zhou, and Koehler 2007). As a result, when RI is interpreted as a predictor of the possibility of reducing spontaneous activity in the central auditory system with acoustic stimulation, it may explain why patients with positive RI in the current study responded more positively to sound enrichment. However, since our study did not have a method that directly assesses activity in auditory and non‐auditory areas, it is not possible to state this with a definitive judgment. The results should be interpreted with caution.

As a result of the present study, THI, VAS, TLL, and MML scores in the RI+ group showed a statistically significant decrease at the end of the post‐treatment sixth month. On the other hand, there is a decrease in the same scores in the RI− group, but this decrease is not statistically significant. Previous studies indicate that the perception of tinnitus severity is influenced by the inhibition of the limbic system at the thalamus level (Besteher et al. 2019; Chen et al. 2017; Golm et al. 2013). This inhibition is related to the extent to which tinnitus is coded with a negative reinforcer in the limbic system. At the end of 6 months, tinnitus in the limbic system may not be coded with negative reinforcement as much as in pre‐treatment. It may even be coded with relatively positive thoughts with the hope brought by the placebo effect, thanks to sound enrichment. As a result, even if the sound enrichment was ineffective and resulted in statistically insignificant decreases in scores, the perception of tinnitus severity may have decreased.

Furthermore, Vanneste et al. (2013) did not find a statistically significant change in the VAS scores of the participants in the study in which they used sound enrichment treatment and found numerically very small differences (∼0.5). One reason could be that participants were not categorized based on their RI status (RI was not assessed in their study). Because many of their participants' tinnitus was characterized by RI−, treatment may have provided limited benefit. Despite the fact that all participants in Vanneste et al. (2013) study were RI−, there are numerically larger differences in the RI− group in our study compared to their study, though these differences are not statistically significant. Vanneste et al. (2013) used sound enrichment treatment for only 1 month in their study. As mentioned in the preceding paragraph, the decreases in the scores in the RI− group in the present study may be attributable to the transformation of negative coding in the limbic system into partial positive coding as a result of the placebo effect.

The tinnitus pitch of the present study participants was ∼7 kHz. Although some previous studies stated that the tinnitus pitch occurs at the audiometric edge (Moore 2010) or at the frequency at which the hearing loss is maximum (Schecklmann et al. 2012), in the current study, the participants' tinnitus was at the frequency where the hearing threshold was around 50 dB, supporting Shekhawat, Searchfield, and Stinear (2014). We attribute this to the discordant theory. Previous studies have found that at 50 dB, outer hair cells are the most damaged and the inner hair cells are the least damaged (Jastreboff 2004). In other words, at 50 dB, the central auditory system's afferent and efferent fibers are most incompatible, so spontaneous activity is at its highest point, and tinnitus occurs at the frequency at this threshold level.

In terms of limitations, we used behavioral test methods in this study to determine whether RI is a predictor that may affect the success of sound enrichment. Imaging and electrophysiological test methods, in addition to these test methods, may be used in future studies to help us better understand the underlying mechanism of RI. Additionally, as the authors know the RI results of the participants, although there were significant differences between the groups, this may have caused a bias. Furthermore, as part of the sound enrichment treatment, participants were asked to listen to a sound created specifically for them for a certain duration and at a specific intensity. Although participants reported completing these durations with the specified intensity, no actual monitoring of sound enrichment usage time and intensity were performed. On the other hand, although there was a significant decrease in THI, VAS, MML, and TLL scores in the RI+ group until the end of the sixth month, this decrease may be related to the natural habituation process that is likely to occur at the end of 6 months (Simões et al. 2021). Therefore, the results should be interpreted with caution. Future studies can take these limitations into account and adjust their methods and group selection accordingly.

To our knowledge, this is the first study to show that the RI phenomenon may play an important role in the success of sound enrichment treatment. RI can be used in the evaluation of tinnitus in clinics. However, there has yet to be an agreement on how the RI should be used in the assessment and treatment of tinnitus or how its results should be interpreted. This study suggests that RI may predict the prognosis of tinnitus treatments used in clinics to reduce the spontaneous firing rate of neurons in the central auditory system. It additionally concludes that RI positivity may predict treatment success in sound enrichment.

## Author Contributions

**Eser Sendesen**: investigation, writing–review and editing, writing–original draft, data curation, resources, methodology, conceptualization. **Didem Turkyilmaz**: project administration, supervision.

## Conflicts of Interest

The authors declare no conflicts of interest.

### Peer Review

The peer review history for this article is available at https://publons.com/publon/10.1002/brb3.70083.

## Data Availability

The data that support the findings of this study are available from the corresponding author upon reasonable request.

## References

[brb370083-bib-0001] Aksoy, S. , Y. Firat , and R. Alpar . 2007. “The Tinnitus Handicap Inventory: A Study of Validity and Reliability.” International Tinnitus Journal 13, no. 2: 94. 18229787

[brb370083-bib-0002] Albert, A. , F. Cangemi , and M. Grice . 2018. “Using Periodic Energy to Enrich Acoustic Representations of Pitch in Speech: A Demonstration.” Proceedings Speech Prosody 2018: 804–808. 10.21437/SpeechProsody.2018-162.

[brb370083-bib-0003] Besteher, B. , C. Gaser , D. Ivanšić , O. Guntinas‐Lichius , C. Dobel , and I. Nenadić . 2019. “Chronic Tinnitus and the Limbic System: Reappraising Brain Structural Effects of Distress and Affective Symptoms.” NeuroImage: Clinical 24: 101976. 31494400 10.1016/j.nicl.2019.101976 PMC6734051

[brb370083-bib-0004] Biswas, R. , A. Lugo , M. A. Akeroyd , W. Schlee , S. Gallus , and D. A. Hall . 2022. “Tinnitus Prevalence in Europe: A Multi‐Country Cross‐Sectional Population Study.” The Lancet Regional Health—Europe 12: 100250. 10.1016/j.lanepe.2021.100250. 34950918 PMC8671623

[brb370083-bib-0005] Chen, Y. C. , W. Xia , H. Chen , et al. 2017. “Tinnitus Distress Is Linked to Enhanced Resting‐State Functional Connectivity From the Limbic System to the Auditory Cortex.” Human Brain Mapping 38, no. 5: 2384–2397. 28112466 10.1002/hbm.23525 PMC6866871

[brb370083-bib-0006] Clarke, N. A. , H. Henshaw , M. A. Akeroyd , B. Adams , and D. J. Hoare . 2020. “Associations Between Subjective Tinnitus and Cognitive Performance: Systematic Review and Meta‐Analyses.” Trends in Hearing 24: 2331216520918416. 10.1177/2331216520918416. 32436477 PMC7243410

[brb370083-bib-0007] Galazyuk, A. , R. Longenecker , S. Voytenko , I. Kristaponyte , and G. Nelson . 2019. “Residual Inhibition: From the Putative Mechanisms to Potential Tinnitus Treatment.” Hearing Research 375: 1–13. 30822633 10.1016/j.heares.2019.01.022

[brb370083-bib-0008] Galazyuk, A. , S. Voytenko , and R. Longenecker . 2017. “Long‐Lasting Forward Suppression of Spontaneous Firing in Auditory Neurons: Implication to the Residual Inhibition of Tinnitus.” Journal of the Association for Research in Otolaryngology 18: 343–353. 27832500 10.1007/s10162-016-0601-9 PMC5352609

[brb370083-bib-0009] Golm, D. , C. Schmidt‐Samoa , P. Dechent , and B. Kröner‐Herwig . 2013. “Neural Correlates of Tinnitus Related Distress: An fMRI‐Study.” Hearing Research 295: 87–99. 10.1016/j.heares.2012.03.003 22445697

[brb370083-bib-0039] Gürses, E. , S. Ercan , M. D. Türkyılmaz , and S. Aksoy (2018). Tinnituslu bireylerde dinleme eforunun değerlendirilmesi: Bir ön çalışma. Türk Odyoloji ve İşitme Araştırmaları Dergisi, 1(1), 15–20.

[brb370083-bib-0010] Haider, H. F. , S. F. Ribeiro , C. Martins , et al. 2020. “Tinnitus, Hearing Loss and Inflammatory Processes in an Older Portuguese Population.” International Journal of Audiology 59, no. 5: 323–332. 10.1080/14992027.2019.1698775. 31829778

[brb370083-bib-0011] Han, B. I. , H. W. Lee , T. Y. Kim , J. S. Lim , and K. S. Shin . 2009. “Tinnitus: Characteristics, Causes, Mechanisms, and Treatments.” Journal of Clinical Neurology 5, no. 1: 11. 19513328 10.3988/jcn.2009.5.1.11 PMC2686891

[brb370083-bib-0012] Herrmann, B. , and B. E. Butler . 2021. “Hearing Loss and Brain Plasticity: The Hyperactivity Phenomenon.” Brain Structure and Function 226, no. 7: 2019–2039. 34100151 10.1007/s00429-021-02313-9

[brb370083-bib-0013] Hu, S. , L. Anschuetz , D. A. Hall , M. Caversaccio , and W. Wimmer . 2021. “Susceptibility to Residual Inhibition Is Associated With Hearing Loss and Tinnitus Chronicity.” Trends in Hearing 25: 2331216520986303. 33663298 10.1177/2331216520986303 PMC7940720

[brb370083-bib-0014] Jastreboff, P. J. 2004. “The Neurophysiological Model of Tinnitus.” In R. M. Aage , L. Berthold , D. Dirk , K. Tobias Tinnitus: Theory and Management, 96–107.

[brb370083-bib-0015] Kaltenbach, J. A. 2006. “Summary of Evidence Pointing to a Role of the Dorsal Cochlear Nucleus in the Etiology of Tinnitus.” Acta Oto‐Laryngologica 126, no. sup556: 20–26. 17114138 10.1080/03655230600895309

[brb370083-bib-0016] King, R. O. , G. S. Shekhawat , C. King , E. Chan , K. Kobayashi , and G. D. Searchfield . 2021. “The Effect of Auditory Residual Inhibition on Tinnitus and the Electroencephalogram.” Ear and Hearing 42, no. 1: 130–141. 32769434 10.1097/AUD.0000000000000907

[brb370083-bib-0017] Levine, R. A. , and Y. Oron . 2015. “Tinnitus.” In J. A. Michael , B. François , F. S. Dick Handbook of Clinical Neurology Vol. 129: 409–431. 10.1016/B978-0-444-62630-1.00023-8 25726282

[brb370083-bib-0018] Moore, B. C. 2010. “The Relationship Between Tinnitus Pitch and the Edge Frequency of the Audiogram in Individuals With Hearing Impairment and Tonal Tinnitus.” Hearing Research 261, no. 1‐2: 51–56. 20103482 10.1016/j.heares.2010.01.003

[brb370083-bib-0019] Newman, C. W. , G. P. Jacobson , and J. B. Spitzer . 1996. “Development of the Tinnitus Handicap Inventory.” Archives of Otolaryngology–Head & Neck Surgery 122, no. 2: 143–148. 8630207 10.1001/archotol.1996.01890140029007

[brb370083-bib-0020] Noreña, A. J. , and J. J. Eggermont . 2005. “Enriched Acoustic Environment After Noise Trauma Reduces Hearing Loss and Prevents Cortical Map Reorganization.” Journal of Neuroscience 25, no. 3: 699–705. 15659607 10.1523/JNEUROSCI.2226-04.2005 PMC6725313

[brb370083-bib-0021] Noreña, A. J. , and J. J. Eggermont . 2006. “Enriched Acoustic Environment After Noise Trauma Abolishes Neural Signs of Tinnitus.” NeuroReport 17, no. 6: 559–563. 16603911 10.1097/00001756-200604240-00001

[brb370083-bib-0022] Ouda, L. , O. Profant , and J. Syka . 2015. “Age‐Related Changes in the central Auditory System.” Cell and Tissue Research 361: 337–358. 25630878 10.1007/s00441-014-2107-2

[brb370083-bib-0023] Roberts, L. E. 2007. “Residual Inhibition.” Progress in Brain Research 166: 487–495. 17956813 10.1016/S0079-6123(07)66047-6

[brb370083-bib-0024] Salazar, J. W. , K. Meisel , E. R. Smith , A. Quiggle , D. B. McCoy , and M. R. Amans . 2019. “Depression in Patients With Tinnitus: A Systematic Review.” Otolaryngology–Head and Neck Surgery 161, no. 1: 28–35. 10.1177/0194599819835178. 30909841 PMC7721477

[brb370083-bib-0025] Schecklmann, M. , M. Pregler , P. M. Kreuzer , et al. 2015. “Psychophysiological Associations Between Chronic Tinnitus and Sleep: A Cross Validation of Tinnitus and Insomnia Questionnaires.” BioMed Research International 2015: 461090. 26583109 10.1155/2015/461090 PMC4637028

[brb370083-bib-0026] Schecklmann, M. , V. Vielsmeier , T. Steffens , M. Landgrebe , B. Langguth , and T. Kleinjung . 2012. “Relationship Between Audiometric Slope and Tinnitus Pitch in Tinnitus Patients: Insights Into the Mechanisms of Tinnitus Generation.” PLoS ONE 7, no. 4: e34878. 10.1371/journal.pone.0034878. 22529949 PMC3329543

[brb370083-bib-0027] Schoisswohl, S. , M. Schecklmann , B. Langguth , W. Schlee , and P. Neff . 2021. “Neurophysiological Correlates of Residual Inhibition in Tinnitus: Hints for Trait‐Like EEG Power Spectra.” Clinical Neurophysiology 132, no. 7: 1694–1707. 10.1016/j.clinph.2021.03.038. 34038848

[brb370083-bib-0028] Seki, S. , and J. J. Eggermont . 2003. “Changes in Spontaneous Firing Rate and Neural Synchrony in Cat Primary Auditory Cortex After Localized Tone‐Induced Hearing Loss.” Hearing Research 180, no. 1–2: 28–38. 12782350 10.1016/s0378-5955(03)00074-1

[brb370083-bib-0029] Sendesen, E. , and D. Turkyilmaz . 2024. “Investigation of the Effectiveness of Sound Enrichment in the Treatment of Tinnitus Due to Hearing Loss.” Brain and Behavior 14, no. 5: e3520. 38715412 10.1002/brb3.3520 PMC11077254

[brb370083-bib-0038] Sendesen, E. (2023). Tinnitus hastalarında rezidüel inhibisyon varlığı ses zenginleştirme terapisinde başarı ölçütü olabilir mi? bir ön çalışma. Türk Odyoloji ve İşitme Araştırmaları Dergisi, 6(3), 98–101.

[brb370083-bib-0030] Shekhawat, G. S. , G. D. Searchfield , and C. M. Stinear . 2014. “The Relationship Between Tinnitus Pitch and Hearing Sensitivity.” European Archives of Oto‐Rhino‐Laryngology 271, no. 1: 41–48. 10.1007/s00405-013-2375-6. 23404467

[brb370083-bib-0031] Sheppard, A. , C. Stocking , M. Ralli , and R. Salvi . 2020. “A Review of Auditory Gain, Low‐Level Noise and Sound Therapy for Tinnitus and Hyperacusis.” International Journal of Audiology 59, no. 1: 5–15. 10.1080/14992027.2019.1660812. 31498009

[brb370083-bib-0032] Shore, S. , J. Zhou , and S. Koehler . 2007. “Neural Mechanisms Underlying Somatic Tinnitus.” Progress in Brain Research 166: 107–548. 17956776 10.1016/S0079-6123(07)66010-5 PMC2566901

[brb370083-bib-0033] Simões, J. P. , P. K. A. Neff , B. Langguth , W. Schlee , and M. Schecklmann . 2021. “The Progression of Chronic Tinnitus Over the Years.” Scientific Reports 11, no. 1: 4162. 10.1038/s41598-021-83068-5. 33602995 PMC7892997

[brb370083-bib-0034] Vanneste, S. , M. van Dongen , B. De Vree , et al. 2013. “Does Enriched Acoustic Environment in Humans Abolish Chronic Tinnitus Clinically and Electrophysiologically? A Double Blind Placebo Controlled Study.” Hearing Research 296: 141–148. 23104014 10.1016/j.heares.2012.10.003

[brb370083-bib-0035] Wang, T.‐C. , T.‐Y. Chang , R. Tyler , et al. 2020. “Noise Induced Hearing Loss and Tinnitus—New Research Developments and Remaining Gaps in Disease Assessment, Treatment, and Prevention.” Brain Sciences 10, no. 10: 732. 33066210 10.3390/brainsci10100732 PMC7602100

[brb370083-bib-0036] Yakunina, N. , and E.‐C. Nam . 2021. “Does the Tinnitus Pitch Correlate With the Frequency of Hearing Loss?” Acta Oto‐Laryngologica 141, no. 2: 163–170. 33146043 10.1080/00016489.2020.1837394

[brb370083-bib-0037] Zhao, Y. , Q. Song , X. Li , and C. Li . 2016. “Neural Hyperactivity of the central Auditory System in Response to Peripheral Damage.” Neural Plasticity 2016: 2162105. 26881094 10.1155/2016/2162105 PMC4736999

